# A reference metagenome sequence of the lichen *Cladonia rangiformis*

**DOI:** 10.1186/s12915-025-02428-z

**Published:** 2025-10-22

**Authors:** Matthias Heuberger, Carlotta Marie Wehrkamp, Alina Pfammatter, Manuel Poretti, Johannes Peter Graf, Aline Herger, Jonatan Isaksson, Edith Schlagenhauf, Rosmarie Honegger, Thomas Wicker, Alexandros G. Sotiropoulos

**Affiliations:** 1 Department of Plant and Microbial Biology, University of Zurich, Zurich, Switzerland; 2 Melbourne Integrative Genomics, University of Melbourne, Melbourne, Australia; 3 Centre for Crop Health, University of Southern Queensland, Toowoomba, Australia

**Keywords:** Metagenome, Bioinformatics, Transposable element, *Asterochloris mediterranea*, Chromosome-scale assembly, *Cladonia rangiformis*, Lichens, Symbiosis, Microbiome

## Abstract

**Background:**

Lichens are an ancient symbiosis comprising the thalli of lichen-forming fungi, their photoautotrophic partners, and their microbiome. So far, they were poorly studied at the genome sequence level. Here, we present a reference metagenome for the holobiont of *Cladonia rangiformis*, aiming to illuminate the genomic complexity and evolutionary interactions within lichen symbioses.

**Results:**

Using long-read sequences from an entire symbiotic complex, plus short-read libraries from 28 additional diverse European lichen samples, we were able to separate genome sequences of 20 individual species. We constructed chromosome-scale assemblies of the *C. rangiformis* fungus and its trebouxioid green algal photobiont *Asterochloris mediterranea*. The genome of the fungus comprises ~ 22% transposable elements and is highly compartmentalized into genic regions and large TE-derived segments which show extensive signatures of repeat-induced point mutations (RIP). We found that *A. mediterranea* centromeres are predominantly derived from two interacting retrotransposon families. We also identified strong candidates for genes that were horizontally transferred from bacteria to both alga and fungus. Furthermore, we isolated 18 near-complete bacterial genomes, of which 13 are enriched in the lichen compared to surrounding soil. Analysis of gene content in fungus, algae, and bacteria identified 22 distinct biosynthetic gene cluster categories for known secondary metabolites.

**Conclusions:**

Our findings revealed that the thalli of *C. rangiformis* have a highly complex microbiome, comprising a mix of species that may include opportunists, ecologically obligate symbionts and possibly even lichen-beneficial bacteria. This study provides the first chromosome-scale genomic framework for a lichen holobiont, offering a foundational resource for future research into metagenomics, symbiosis, and microbial ecology in lichens.

**Supplementary Information:**

The online version contains supplementary material available at 10.1186/s12915-025-02428-z.

## Background

Lichens are the symbiotic phenotype of a polyphyletic group of nutritional specialists among asco- and basidiomycetes (fungi) which acquire fixed carbon in a mutualistic symbiosis from a population of cyanobacterial or green algal cells, rarely of both; these are incorporated in the fungal thallus [33, 74]. Species names of lichens refer to the fungal partner, i.e., the quantitatively dominant mycobiont,the photobiont has its own name and phylogeny. Lichenization is an ancient, ecologically successful nutritional strategy, going back at least to the lower Devonian (420 My, [36]), approx. 17% of extant fungi being lichenized [51]. Lichen thalli were defined as consortia with an unknown number of participants [33] or as complex ecosystems [30].

*Cladonia* lichens play an important role as a widespread genus found in all continents, including Antarctica [19]. Some of these lichens can be quite sturdy and are found in exceptionally degraded environments such as in contaminated slag dumps [63]. However, sometimes these lichens show sensitivity and might be under threat from human activities [53, 79]. Among the various uses of *Cladonia* species, one important facet is related to their nutritional values as they are consumed by animals such as reindeer and thus are of importance to rural communities like that of the Sami people [2]. At the same time, many *Cladonia* species have been used for their antimicrobial and other medicinal capabilities in the past [2]. Their components and products are currently being researched by the global scientific community [1, 43].

Morphologically advanced lichens have internally stratified thalli where photobiont cells are placed in optimal positions regarding illumination and gas exchange [35]. The false reindeer lichen *Cladonia rangiformis* has a massive tubular fungal axis, the podetium, which branches abundantly in its terminal, youngest parts. The green algal photobionts (*Asterochloris mediterranea* or other *Asterochloris* spp., [58]) are kept in islets at the periphery of the podetia below a thin cortex. In the fruticose *C. arbuscula*, large numbers of bacteria grow on the inner surface of the tubular podetium and between thick-walled, glucan-rich conglutinate hyphae of the fungal axis (Figs. 6.5.g–j in [34]). Like true reindeer lichens, *C. rangiformis* rarely reproduces sexually, pycnidia, and ascomata being formed at the tip of the youngest branches. The thalli of reindeer lichens are elastic when wet, but very brittle when dry and in this state prone to fragmentation, e.g., by trampling. Fragments containing the mycobiont and photobiont and their microbiome are successfully dispersed by wind, animals, or humans. A possible intercontinental anthropogenic dispersal of the West European *C. rangiformis* was described [21].

Diversity of the lichen microbiome is often assessed by using operational taxonomic units (OTUs) as proxy for species. Bacteria are typically classified based on 16S rDNA sequences, where samples with ~ 98% sequence identity are defined as belonging to the same species, while ~ 96% identity places them in the same genus. Eukaryotes species are routinely defined through internal transcribed spacers (ITS) sequences of rDNA, where samples of the same species usually share > 98% ITS sequence identity [23, 38]. However, OTU counts should be considered carefully because sequence identity cutoffs may vary between studies. For example, a study on metagenomes of *Cladonia* lichens identified 79 different ITS2 OTUs, ~ 81% were fungi, and 18% algae [78]. However, most of the fungal sequences originated from only four individual *Cladonia* species, while most green algae OTUs could be assigned to *A. mediterranea*.

Shishido and colleagues [78] identified 158 OTUs for bacterial 16S rDNAs, most belonging to *Alphaproteobacteria* and *Acidobacteria*. Similar findings were made in the Antarctic lichen *Cladonia squamosa* [62] where most of the ~ 800 OTUs belonged to the *Alphaproteobacteria* and *Acidobacteria*, with *Rhodospirillales* and *Rhizobiales* being the most abundant orders. In *C. arbuscula*, *Acetobacteraceae* closely related to the genera *Gluconacetobacter*, *Acidisphaera*, and the species *Rhodovastum atsumiense* were found [15]. However, the microbiome of *Cladoniaceae* may also vary depending on the environment [67], or even along the axis of single thalli [89].

Studies that aim at unraveling entire lichen metagenomes are rare. The main challenge working with metagenome assemblies is that they comprise a complex mixture of thousands of sequence contigs from fungi, algae, and bacteria, which have to be assigned to individual species. Genomes reconstructed from metagenomes are referred to as metagenome-assembled genomes (MAGs). Here, taxon-specific characteristics such as GC content, oligonucleotide frequencies, and gene content can be used to infer to which species contigs belong. Additionally, there are specialized softwares such as MetaBAT2 [41] or CONCOCT [4] that use sequence coverage and composition information to group contigs into “bins “ of different organisms which ideally represent complete MAGs.

The first lichen metagenome published was from *Umbilicaria pustulata* [86], using a combination of PacBio long reads and Illumina short reads. The genome of the *U. pustulata* fungus has a size of 33 Mb, while the *Trebouxia* sp. photobiont has a 53 Mb genome. The study found that the two organisms were present at a relative abundance of fungal to algal nuclei of ∼20:1. The bacterial community was characterized through identification of sequence contigs containing 16S rDNA sequences. It was dominated by *Acidobacteriaceae* and contained bacteria similar to those typically found in lichens (e.g., *Lichenibacterium*, [64, 65]). Very recently, Tagirdzhanova et al. [83] re-examined 400 publicly available lichen metagenome sequences for their organismal content, leading to the assembly of nearly 1000 MAGs. They identified most of the retrieved algal MAGs to belong to the genus *Trebouxia* and in addition found four bacterial families, which were specifically abundant across the studied metagenomes: *Acetobacteraceae*, *Acidobacteriaceae*, *Beijerinckiaceae*, and *Sphingomonadaceae*.

Genome sequences for *Cladonia* lichens are rare. We found genome assemblies for only nine *Cladonia* fungi (Additional file 1: Table S1), four of which are assembled into (near) chromosome-scale scaffolds, while the other five are fragmented into hundreds or even thousands of sequence contigs. One reason for the fragmented assemblies may be repeat-induced point mutations (RIP), a fungal mechanism to silence transposable elements (see below). The publicly available *Cladonia* fungi genomes are 32–39 Mb in size, which is rather typical for fungi (mycocosm.jgi.doe.gov/mycocosm/home/1000-fungal-genomes). Additionally, we are aware of only four published algae genomes from the *Asterochloris* genus (Additional file 1: Table S1.). Furthermore, a few genomes from bacteria that commonly live in *Cladonia* lichens have been assembled: *Lichenibacterium minor*, *Lichenibacterium ramalinae*, and *Lichenicoccus roseus* [64, 65].

Considering the limited data on metagenome sequences in lichens in general and *Cladonia* in particular, we aimed at producing a reference metagenome for *C. rangiformis* that includes fungus, alga, and bacteria. We report here on the composition of the chromosome-scale assemblies of the *C. rangiformis* fungus and its photobiont *A. mediterranea*. Additionally, we present MAGs for 18 bacterial species.

## Results and discussion

The *Cladonia* sample GR013, which was collected on the Sithonia peninsula of Chalkidiki, was chosen to generate the reference genome. The sample was whole metagenome sequenced using PacBio long reads and Illumina short reads. The primary PacBio assembly of the *C. rangiformis* reference sample yielded 2707 sequence contigs. Nine contigs were identified as miss-assembled hybrids and broken up accordingly (Additional file 2: Additional file 2: Fig. S1, methods) resulting in an assembly of 2716 contigs with a cumulative length of 226.5 Mb. This primary assembly is a mixture of sequences from fungi, algae, and bacteria which had to be assigned to individual species. To facilitate the species assignment, we also sequenced metagenomes of 28 additional lichen samples from five European countries (Greece, France, Finland, Iceland,, and Switzerland, Additional file 1: Table S2) with Illumina short read technology. Since the main focus of this study were *Cladonia* lichens, 16 of the samples represent this genus, but more distantly related lichens were also included (Additional file 1: Table S2). The rationale for having these additional samples was to have quantitative sequence read coverage data from which the abundance of individual species in the respective samples can be inferred (i.e., species with higher abundance will be represented with higher number of sequence reads in the Illumina data). This data from the additional lichen samples proved essential in defining and isolating individual bacterial genomes in the reference *C. rangiformis* (see below).

### Separation of main groups of organisms in the *C. rangiformis* metagenome assembly

**Fig. 1:**
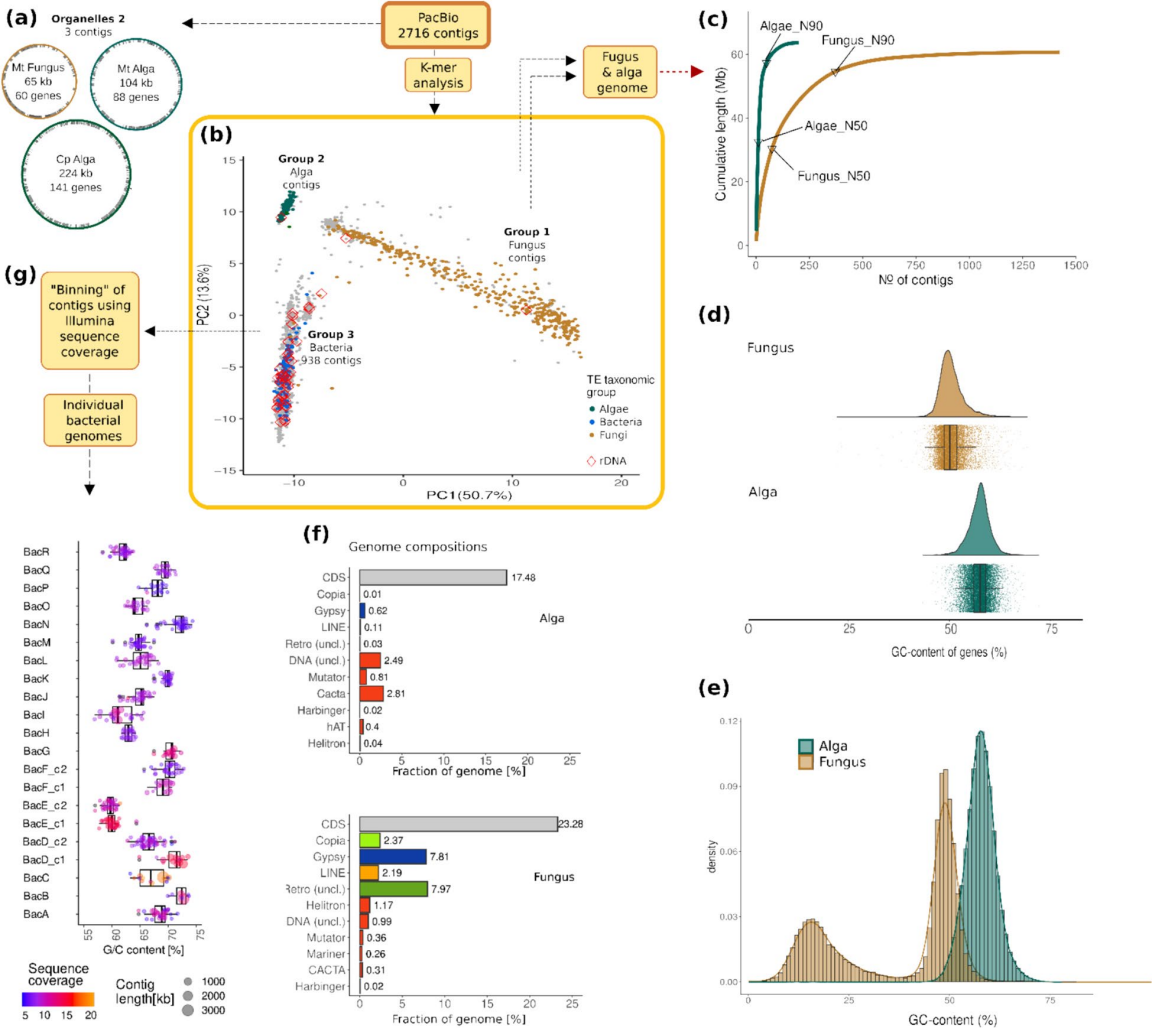
Separation of genomes from individual species from the *C. rangiformis* metagenome (a detailed workflow is shown in Additional file 2: Fig. S2). **a** Separation of eukaryotic organelles. **b** Separation of sequence contigs into fungal, algal, and bacterial contigs based on tetramer frequencies. **c** Genome coverage by sequence contigs ordered by their size. The alga fraction consists mostly of very large sequence contigs, while the fungal fraction is more fragmented. **d** GC content of coding regions (CDS) of fungal and algal genes. **e** GC content of 500 bp windows from the fungus and alga genomes. **f** Gene and TE content of the fungus and alga genomes. **g** Bacterial sequence contigs were further divided into “bins,” which ideally represent complete bacterial genomes (see the “Results” section)

To isolate genomes of individual species, we used multiple steps and criteria to separate contigs into “bins,” which ideally represented genomes of individual species. The simplified workflow is shown in Fig. 1 (more detailed in Additional file 2: Fig. S2). First, we isolated the mitochondria and chloroplast genomes which were assembled in single contigs (Fig. 1a, Additional file 2: Fig. S3). We then classified the remaining sequence contigs into the three main organism groups, fungi, algae, and bacteria using k-mer frequencies. Here, frequencies of all 256 possible tetranucleotides were calculated for all 2716 sequence contigs. We chose this approach because initial tests showed that k-mer frequencies are a surprisingly efficient way to distinguish sequences from fungi, algae, and bacteria, which would also reduce complexity in the subsequent separation of bacterial genomes and reduce the risk of contamination of bacterial assemblies with eukaryotic sequences. For initial identification of the main groups, we only used contigs larger than 10 kb, because the species-specific tetramer frequencies can be poorly represented in shorter contigs. Principal component analysis (PCA) clearly segregated three distinct groups of contigs (group 1, 2, and 3, Fig. 1b, Additional file 2: Fig. S4). Group 1 contigs showed a wide range in GC content (Additional file 2: Fig. S4b), indicating that it represents the fungal genome. In fungal genomes, we expected gene-containing regions to have higher GC content, while TE-derived sequences have very low GC content due to RIP (see below).

To assign contigs to fungi, algae, and bacteria, we also searched the contigs for sequences encoding canonical TE proteins such as transposase, reverse transcriptase, or integrase. This confirmed group 1 contigs to represent the fungal genome, as they encode proteins with highest similarity to fungal retrotransposon superfamilies. Group 2 contigs contained numerous sequences with homology to plant TEs and thus represent the algal genome. Group 3 contigs were classified as representing bacteria genomes, because many encoded proteins with homology to typical class 2 (DNA) transposons from bacteria, while retrotransposons were absent (Fig. 1b, S4c).

The 1034 contigs shorter than 10 kb were assigned to the 3 main groups in a second step (Additional file 2: Fig. S2). Here, we again took advantage of the fact that eukaryotic TEs are highly species specific. Therefore, we assigned sequence contigs that contain TE sequences to either fungus or alga based on DNA sequence homology: we used the contigs < 10 kb as queries in blastn searches against the sequence contigs > 10 kb that were assigned to the alga and the fungus, respectively, in the previous step. Additionally, we identified fungal contigs based on signatures of repeat-induced point mutations (RIP, see the “Methods” section). This allowed us to assign 847 of the contigs < 10 kb to the fungus and 39 to the algal pool.

The 937 contigs classified as coming from bacteria were further subdivided separately with the goal to identify the genomes of individual bacterial species (Fig. 1g, see below).

Additionally, rDNA sequences identified in 41 contigs (38 bacterial, one algal, and two fungal contigs) were also mapped onto the PCA (Fig. 1b. Additional file 2: Fig. S4d). Using the ITS sequences for blastn searches against the NCBI databases, we determined the fungus to be of the species *C. rangiformis* and the alga *A. mediterranea*. Previous studies reported the presence of lichenicolous or endolichenic fungi in lichen holobionts (e.g., *Capnobotryella*, *Cladophialophora*, *Coniosporium*, *Mycosphaerella*, and *Rhinocladiella* in [7, 29],*Cladophialophora* in [17],*Didymocyrtis cladoniicola* and *Epicladonia simplex* in [54]). However, we did not find any ITS sequences belonging to fungi other than *Cladonia*. Nevertheless, we found an ITS belonging to *C. humilis* at a very low sequence coverage (~ 250-fold less than *C. rangiformis)*, suggesting the presence of a *C. humulis* fungus at low abundance.

When analyzing sequence coverage of the 1420 sequence contigs from fungi, we found that our sample likely consisted of two haplotypes of *C. rangiformis*, with the predominant haplotype making up at least 80% of the sample. Multiple haplotypes were previously found in two *Cladonia* genome sequences, *Cladonia norvegica* (Genbank accession GCA_963971275.1 alternate haplotype with GCA_963971245.1 as the principal haplotype) and *Cladonia squamosa* (Genbank accession GCA_947623575.2 alternate haplotype with GCA_947623385.2 as the principal haplotype) (Additional file 1: Table S1).

The predominant haplotype was assembled in 151 large contigs with an Illumina read coverage of ~ 220 (Fig S3, S5). We anchored these to a recently published high-quality assembly of *C. squamosa* (Genbank accession GCA_947623385.2) and produced a chromosome-scale assembly with 23 chromosomes and a total size of 34 Mb. The remaining 1269 contigs were overall much shorter and had much lower read coverage (Additional file 2: Fig. S5a). Most were classified as coming from the second haplotype, while a small number may represent other fungi that were present at very low abundance (Additional file 2: Fig. S5a). These 1269 contigs were collected in a separate bin and not further analyzed.

A total of 11,782 protein-coding genes were annotated on the 23 chromosomes, with 98.3% of gene models having an AED (annotation edit distance) measure below 0.5. The assembled fungal genome has a very high BUSCO (Benchmarking Universal Single-Copy Orthologs, [52, 80]) score of > 96% (Additional file 2: Fig. S5c). The *C. rangiformis* coding sequences (CDS) have a median GC content of 50.4%, similar to those of other Ascomycetes (Fig. 1d, Additional file 2: Fig. S5d). Additionally, the *C. rangiformis* genome contains ~ 22% TEs. Most are retrotransposons, while only few DNA transposons were found (Fig. 1f). The *C. rangiformis* fungus is enriched in genes for immune response and secondary metabolite production.

To identify gene families that are specifically expanded in lichens, we compared gene contents of the *C. rangiformis* fungus and three other lichen fungi (*Letharia lupina*, *Ramalina farinacea*, and *Xanthoria parietina*) with that of *Neurospora crassa*, an established model fungus which does not form lichens, but has strong RIP activity [12, 13]. We found the lichen genomes to be highly enriched in genes encoding Ankyrin repeats, with *C. rangiformis* showing the highest numbers (Additional file 2: Fig. S6). Ankyrin repeat-containing proteins were suggested to be involved in immune response and/or non-self-recognition [84]. Additionally, the four lichen genomes show high enrichment in Cytochrome P450 genes which are involved in the production of lichen-specific secondary metabolites [57]. Furthermore, several enriched families encode non-ribosomal peptide synthases for siderophores such as Enterobactin (Additional file 2: Fig. S6), which are highly effective in the mobilization of metal ions [16].

### The genome of the *C. rangiformis* fungus shows strong signatures of repeat-induced point mutations

Most notable, when the *C. rangiformis* genome is analyzed in 500 bp windows, it shows a bimodal GC content distribution (Fig. 1e) with gene-containing regions showing a relatively narrow peak at ~ 48%, while TE-derived sequences have much lower GC content with a local peak at ~ 16% (Fig. 1e), indicating high RIP activity. RIP highly efficiently silences TEs through conversion of C- > T during sexual reproduction, resulting in repetitive sequences being depleted in G and C content [12, 56]. Indeed, we found homologs of *RID* and *DIM-2*, the proposed core genes of the RIP pathway [25]. From the local minimum of the GC content distribution, we defined sequences with a GC content lower than 35% as affected by RIP (“ripped”) and those with higher GC content as “unripped.” Using this threshold, 62.5% of 500 bp genomic windows were counted as unripped and 37.5% as ripped. Similarly high levels of RIP were reported in only a few other fungi such as *Leptosphaeria maculans (syn. Plenodomus lingam)*, while most other RIP-containing ascomycetes show weaker signatures [5]. Indeed, our own analysis of 112 fungal genomes identified only 5 ascomycete species with similarly strong RIP signatures (Additional file 2: Fig. S7).

**Fig. 2:**
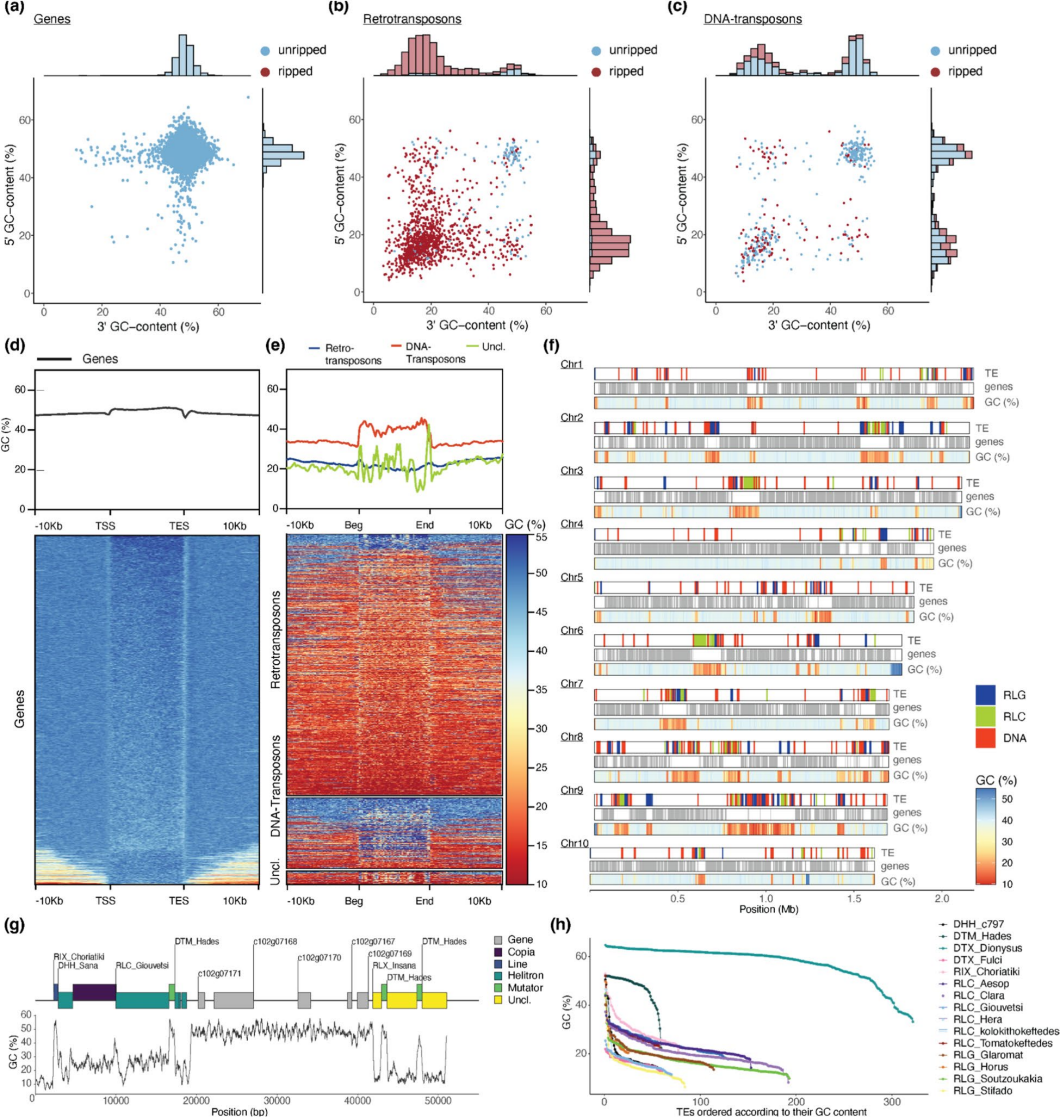
Analysis of signatures of repeat-induced point mutations (RIP) in the *C. rangiformis* genome. **a** GC-content of 1 kb 5’ and 3’ flanking sequences of genes. Dots represent annotated genes with coloring based on whether they have a GC-content of below or above 35%. The histograms along the axes reflect the densities of dots in *x* and *y* direction. **b** Analogous plot for flanking sequences of retrotransposons. **c** Analogous plot for flanking sequences of DNA transposons. **d** GC-content across genes and 10 kb 5’ and 3’ flanking sequence calculated in 200 bp windows. Gene lengths from transcription start (TSS) to end site (TES) were normalized. The top panel shows the average values across all annotated genes, while the bottom shows a heatmap where each horizontal line corresponds to a single gene and its flanking sequences. **e** Analogous plot for transposable elements with retrotransposons, DNA transposons, and unclassified TEs treated separately. **f** RIP signatures in large sequence contigs. The two top tracks show distributions of TE and genes, respectively, while the bottom track shows the GC content in 1 kb windows as a heat map. **g** An annotated example of a sequence contig containing ripped and unripped regions. TEs are shown as colored boxes, where nested TEs are shown raised above those into which they have inserted. Note that TEs that inserted later often have higher GC content, indicating that they went through fewer rounds of RIP. **h** GC content of individual copies of the 15 most abundant TE families. TE copies are ordered by their GC content from highest to lowest

To study to what degree genes and TEs are affected by RIP, we analyzed the GC content of genes and TEs, as well as of their flanking regions in 5’ and 3’ direction. As shown in Fig. 2, genes are practically unaffected by RIP, and their flanking sequences have in most cases roughly the same GC content. This is either due to the low-copy nature of protein coding genes, or because RIP in genes is selected against. In contrast, retrotransposons are primarily found in regions of very low GC content (Fig. 2b, e). This could either mean that retrotransposons specifically target “ripped” regions, or that insertions into gene-containing (i.e., “unripped”) segments are effectively selected against. Additionally, the retrotransposons themselves are mostly ripped except for a few copies which were inserted into unripped regions (Fig. 2e). Interestingly, DNA transposons are found in ripped as well as unripped regions, interspersed with genes. Furthermore, the DNA transposons themselves often show no RIP signatures (Fig. 2c, e, f, g, and h). Indeed, some DNA transposon families were probably recently active, as many sequences contain newly inserted TEs which are characterized by a GC content similar to that of genic regions. Examples are the *DTM_Hades* elements shown in Fig. 2g.

The *C. rangiformis* genome is strongly compartmentalized with regard to RIP signatures: most chromosomes have one large 100 to 500 kb RIP region, which we interpret as centromeres (example in Fig. 2f) and a few additional, somewhat smaller, RIP islands. Here again, we found DNA transposons occasionally interspersed with genes in unripped compartments, while retrotransposons are predominantly clustered in ripped regions (Fig. 2f).

The boundary between “gene space” and TEs is usually very sharp (example in Fig. 2g), indicating that TEs are precisely recognized by the RIP machinery. Additionally, there are varying levels of RIP: the majority of TE-derived sequences have a very low GC content of 10–20%, while a fraction has an intermediate GC content of 20–40%. We interpret the latter as TEs that were more recently active and thus went through fewer RIP cycles (for example, the *DHH_Sana* and *RLC_Giouvetsi* elements in Fig. 2g).

Curiously, the two families of DNA transposons identified here (*DTX_Dionysus* and *DTM_Hades*) show generally much lower levels of RIP than retrotransposons, although they are found at roughly even proportions in ripped and not ripped regions (Fig. 2f). Additionally, for retrotransposon families, there are only very few copies that are not ripped, presumably the most recently inserted ones, while all other copies show a rapid decline toward GC content of 10–20% when copies are ordered by their GC content (Fig. 2h). In contrast, *DTM_Hades* and *DTX_Dionysus* show the inverse GC content distribution with most copies having high GC content, while only very few underwent RIP to varying degrees (Fig. 2h). It is possible that these two TE families simply escape RIP because of their small size of ~ 730 bp. Alternatively, it is possible that these two TE families were highly active very recently, and only few copies have been subjected to RIP so far.

### The centromeres of the alga *A. mediterranea* are defined by transposable elements

**Fig. 3:**
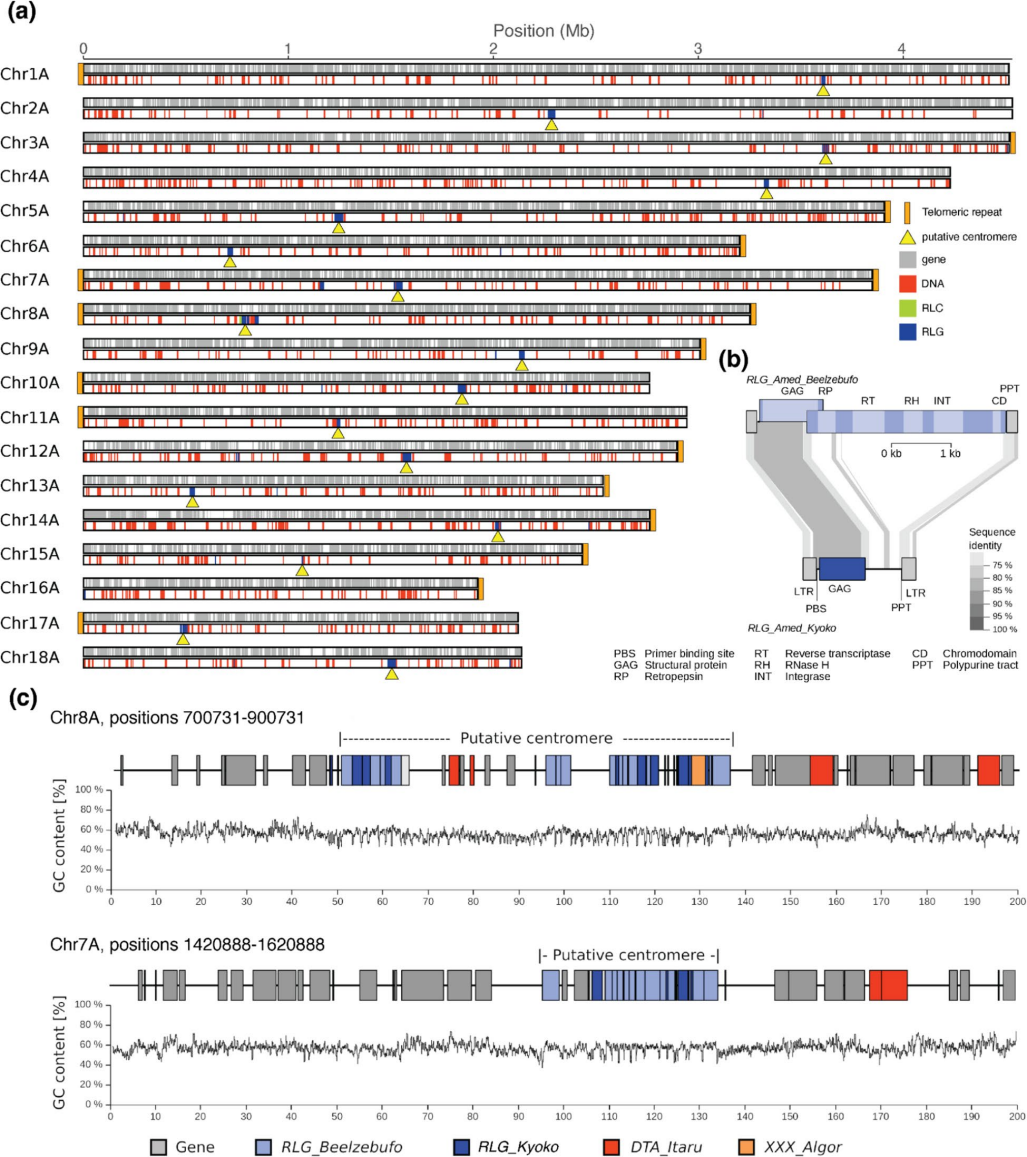
Analysis of chromosome-scale contigs in the *Asterochloris* alga genome.** a** Graphic representation of the 18 chromosome-scale contigs. The top track shows positions of annotated TEs, telomeres, and putative centromeres, while the bottom track indicates positions of annotated genes. **b** Sequence organization of *RLG_Beelzebufo* (top) and *RLG_Kyoko* retrotransposons (bottom). *RLG_Beelzebufo* is a putative autonomous retrotransposon, meaning it encodes all necessary enzymes for its replication, while *RLG_Kyoko* is a non-autonomous derivative which encodes only a GAG protein. Sequences which are conserved at the DNA level are connected with gray areas with darker gray indicating higher sequence identity. **c** Detailed annotation of two putative centromeres

The *A. mediterranea* genome was assembled into much longer contigs than the fungus, despite algal contigs having a much lower sequence coverage of ~ 21 ×. This is likely due to a low repeat content and the absence of RIP, both make contig assembly less problematic. We anchored the algal contigs to a recently published genome sequence of an *Asterochloris* species (genbank accession GCA_963969365.1) and found that we could cover the entire genome with 55 sequence contigs, with 5 contigs representing entire chromosomes. The resulting chromosome-scale assembly has a total size of 57 Mb, in 18 chromosomes, very similar to those coming from algae sequenced from pure cultures (Additional file 1: Table S1). A high level of completeness is indicated by telomeric sequences at most chromosome ends (Fig. 3). The leftover 143 contigs that were classified as coming from algae are short and have lower sequence coverage (Additional file 2: Fig. S5). They likely represent other algae species that were present at very low abundance and were not analyzed further.

Annotation of the *A. mediterranea* genome predicted 9107 protein-coding genes, with 88.8% of gene models having an AED measure below 0.5. BUSCO evaluation of this annotated protein-coding gene set yielded a completeness score of 90.2% ([S:88.9%,D:1.3%,F:1.3%,M:8.5%,N:1519; chlorophyta_odb10). The GC content of *Asterochloris* CDS (median 59.4%) is higher than that of the fungus (Fig. 1e) and at the higher end compared with other plants (Additional file 2: Fig. S5d).

Only ~ 8% of the *A. mediterranea* genome was annotated as TE-derived. Because no genome-wide analysis of the repetitive fraction of algae from lichens has been published so far, we focused our analysis on TE sequences. Along chromosome arms, we found predominantly DNA (class 2) transposons interspersed with genes (Fig. 3).

Interestingly, most chromosomes contain exactly one distinct locus that is highly enriched in LTR retrotransposons, which we interpreted as centromeres (examples in Fig. 3b). Note that we use the term “centromere” here to refer to both functional centromeres and pericentromeric regions, as it is difficult to distinguish the two without epigenetic data such as CENH3 ChIP-seq. We identified two putative centromere-specific LTR retrotransposon families *RLG_Beelzebufo* and *RLG_Kyoko* (Fig. 3b). These were found in all putative centromeres (examples in Fig. 3c) but are practically absent from chromosome arms. Previous studies showed that many plant and fungi species have centromere-specific TEs (e.g., [3, 59, 76]).

The two TE families have an interesting biology: *RLG_Beelzebufo* is a putatively autonomous retrotransposon, meaning it encodes all necessary enzymes for its replication (Fig. 3b). Most importantly, it also encodes a so-called chromodomain that is fused to the integrase enzyme. Such domains are predicted to recognize the centromere-specific histone variant CENH3 and thus may target the insertion of retrotransposon copies into functional centromeres [3, 31, 60]. The second centromere-specific TE family, *RLG_Kyoko*, is a deletion derivative of *RLG_Beelzebufo* which lacks genes necessary for its replication such as the reverse transcriptase and integrase (Fig. 3b). We propose that *RLG_Kyoko* is cross-mobilized by *RLG_Beelzebufo* as the two fulfill the typical criteria previously described for autonomous/non-autonomous TE pairs [90]: First, the LTRs, which contain regulatory sequences, are conserved between the two, suggesting that they are likely co-expressed. Second, diagnostic motifs such as primer binding site and polypurine tract for reverse transcription are identical in the two (Fig. 3b). The situation is very similar to that in centromeres of wheat [31], where the autonomous *RLG_Cereba* and the non-autonomous *RLG_Quinta* retrotransposons are the main components of functional centromeres. Interestingly, this seems to have evolved independently in algae and wheat, since *RLG_Beelzebufo* and *RLG_Cereba* represent different lineages of the same superfamily containing different types of chromodomain, indicating that they acquired these domains independently.

### Isolation of bacterial genomes from the bacteria contig pool

Tetramer analysis identified a pool of 937 putative bacterial PacBio contigs, expected to represent several different bacterial species. Our aim was to group bacterial sequence contigs into “bins”, which ideally represent complete bacterial genomes. To accomplish this, we mapped Illumina sequence data from our 29 lichen samples to the 937 bacterial contigs and calculated the average read coverage for each PacBio contig across all 29 samples.

We assumed the individual bacterial species’ abundance to differ between the different lichen samples, resulting in a sample specific average sequence coverage per contig. At the same time, we expected the sequence contigs belonging to one species to have similar average sequence coverage within a sample. To group contigs into bins, we utilized the previously published software CONCOCT [4] and MetaBAT2 [41]. Additionally, we produced two correlation matrices derived from two different read mapping qualities (0 and 60) for manual binning. The four approaches yielded between 36 and 47 contig bins. There was substantial overlap between contig bins generated with the four approaches, although we also frequently found differences between methods. To consolidate the results, we defined bacterial genomes as groups of contigs that are common to bins from at least two of the four approaches (Additional file 2: Fig. S8). In total, we defined 18 metagenomic bins, comprising 7 to 56 PacBio contigs and sizes ranging from 1.04 to 5.67 Mb. A total of 377 bacterial contigs with a cumulative size of 27.7 Mb remained unassigned (Fig S2).

We assessed the completeness and quality of the retrieved metagenomic bins using BUSCO and CheckM (Additional file 2: Fig. S9). Ten of the bins likely represent near-complete bacterial genomes since three (BacB, BacC, and BacD_chr1) had high BUSCO and CheckM completeness scores of over 85%, while seven showed medium BUSCO completeness scores ranging from 50 to 70% (BacA, BacE_chr1, BacF_chr1, BacG through BacI, and BacK).

Interestingly, BacN, M, O, and P show very low BUSCO and CheckM scores and are also relatively small in size (1–2.6 Mb). However, contigs contained in these were consistently binned together in all four binning approaches (Additional file 2: Fig. S8), which makes us confident of their assignment. We hypothesize that BacM through BacR as well as BacD_chr2, BacE_chr2, and BacF_chr2 represent secondary replicons, so called chromids. Chromids have characteristics from both bacterial chromosomes and plasmids (e.g., plasmid-like replication systems, [28]) and are found in about 10% of bacteria [28]. As chromids contain only few essential core genes, but rather operons for specialized metabolic pathways, this would explain the low completeness scores.

To assess to what coverage a bacterium needs to be sequenced, to be sufficiently well assembled (defined as BUSCO ≥ 75), we mapped the PacBio long reads back onto our metagenome, and compared sequence read coverage of the respective bacterial MAGs to their BUSCO scores (see the “Methods” section). From this analysis, we conclude that there need to be approximately 3000 reads of a length of ~ 15 Kb to sufficiently assemble a genome of 4 Mb (the average size of bacterial MAGs in our metagenome).

We studied the presence of the isolated bacteria genomes in our 29 lichen samples by searching for their 16S rDNA (Additional file 2: Fig. S10). For most, we found at least rDNA from bacteria of the same genus, but they were generally more frequent in *Cladonia* than in other samples (Additional file 2: Fig. S10). None of them was found in all samples, which might indicate that none of them are obligate for the symbiosis. However, it is also possible that the respective rDNA was simply not covered well enough by the sequencing.

### The *C. rangiformis* microbiome comprises at least 23 bacterial species

**Fig. 4:**
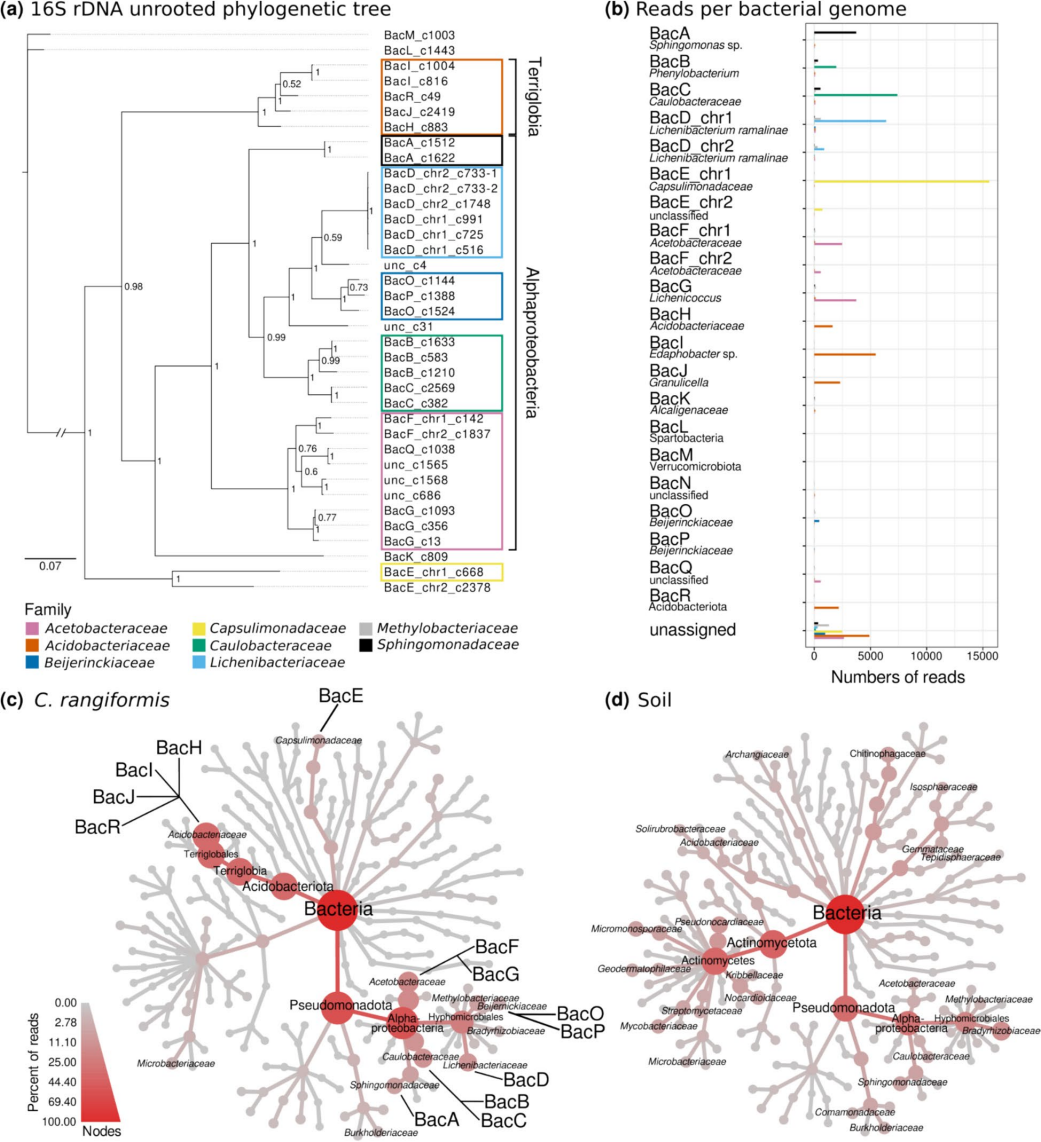
Bacterial genomes identified in the *C. rangiformis* metagenome. **a** Phylogenetic tree of 16S rDNA sequences. The MCMC analysis was run for 80,000 generations with a burn-in value of 25%. The nodes display the probability values. Colored boxes indicate the bacterial family based on panel **b**. 16S sequences retrieved from the metagenome but not assigned to a bacterial genome are labeled as unclassified (unc). **b** Number of reads per bacterial family and identified bacterial genome. The taxonomic classifications of the reads are indicated by the colors of the bars. Unassigned reads are the reads which were classified as one of the represented families by MEGAN but did not map to one of the retrieved bacterial genomes. Further classification is based on hits of the 16S rDNA in the NCBI database (> 98% identity for species, > 96% for genus, and > 90% for family and other taxonomic levels). **c** Taxonomic tree of bacterial families in the reference genome and its corresponding soil sample (**d**). The taxonomic classification was retrieved from the DIAMOND + MEGAN pipeline. Families with incidence in either the lichen or the soil samples were included in the taxonomic trees. The size of nodes and the node color represent the percentage of reads of the taxonomic levels within the respective sample. Bacterial families which were represented in more than 1% of the reads in the respective sample are labeled

We taxonomically classified the bacteria based on their 16S rDNA. In total, we identified 37 sequence contigs that contain 16S rDNA sequences in the 937 bacterial contigs, 32 of them were found in the 18 isolated bacterial genome bins. In bacteria, rDNA operons are found in multiple loci along bacterial chromosomes (unlike in eukaryotes where rDNA genes are usually found in a single locus). Importantly, rDNA genes coming from a single species are usually (nearly) identical due to recurring gene conversion between loci [50]. Indeed, a phylogenetic tree showed that 16S rDNA sequences coming from the same genome consistently clustered together (Fig. 4a), further validating the bacterial genome assemblies. In total, we estimate that our *C. rangiformis* metagenome assembly contains sequences from at least 23 species (Fig. 4a).

Interestingly, only four of the 18 bacteria had matches with at least 98% identity in NCBI, a commonly used cutoff for bacteria to be classified as the same species, indicating that our reference sample contains mainly poorly described bacteria. Nine of our retrieved genomes belong to the class *Alphaproteobacteria*. They could be further classified into families such as *Acetobacteraceae*, *Caulobacteraceae*, and *Sphingomonadaceae* (Fig. 4a and b). BacN which might represent a chromid does not contain an rDNA sequence and therefore remained unclassified.

Of particular interest was BacD, which has a main chromosome (chr1) and a putative chromid (chr2). We are confident that the two chromosomes belong to the same species as all six 16S rDNA sequences found on chr1 and chr2 (3 on each) are identical (Fig. 4a). Its 16S rDNA is 99% identical to that of *Lichenibacterium ramalinae* which was isolated in the subarctic region of Russia [64]. Interestingly, the strain present in our sample is extremely similar to the previously published one, with 41% genes being > 90% identical. It is intriguing to find such closely related bacteria strains in completely different climate zones. Additionally, we found bacteria of the genus *Lichenibacterium* in 19 of our 29 lichen samples (Additional file 2: Fig. S10). Furthermore, the published *L. ramalinae* does not contain a chr2 homolog. In our assembly, chr2 had consistently lower Illumina read coverage across *Cladonia* samples (Additional file 2: Fig. S8), suggesting that it may be present in only a fraction of the bacteria.

Bacterial genome G was classified to belong to the genus *Lichenicoccus* due to > 96% identity to *Lichenicoccus roseus* (Additional file 1: Table S6), a bacterial species isolated from *C. arbuscula* and *C. stellaris* lichens from Russia [65]. We found bacteria of the same genus as BacG in 14 of our 16 *Cladonia* samples from north to south Europe (Additional file 2: Fig. S10), indicating that they are a common part of lichen thalli.

Bacteria I, J, and R belong to the genera *Edaphobacter* (BacI) and *Granulicella* (BacJ, BacK), which were previously described as soil bacteria adapted to arctic climate [42, 71]. Our findings now indicate that they are common in *Cladonia* lichens across Europe, as we found bacteria of these genera in most of our samples (Additional file 2: Fig. S10).

In addition to isolating bacterial genome sequences, we assessed microbiome diversity by taxonomically classifying the Illumina raw reads using the DIAMOND + MEGAN pipeline. Approximately 100,000 reads could be classified at minimum to the bacterial family level, and about 90% of them were from eight families (Additional file 2: Fig. S11). Most of these reads mapped to our 18 bacterial genomes with only about 10% mapping to unassigned bacterial contigs (Fig. 4b). The read mapping validated the bacterial genome assemblies as all of them had predominantly reads from a single family mapped to them (Fig. 4b). Additionally, the classification using MEGAN was consistent with the phylogenetic analysis of the 16S rDNA sequences (Fig. 4a and b). About 4800 reads classified as *Acetobacteriaceae* were not assigned to a genome. We therefore assume the presence of a third bacterial species from the family *Acetobacteriaceae*.

### *C. rangiformis* and its surrounding soil exhibit distinct microbiome profiles

To gain insight which bacteria may be specific to the lichen symbiosis, we compared the microbiome of our lichens with a soil sample collected in the vicinity of the lichen thalli. The profile of bacterial families present in both samples differs considerably (Fig. 4c, d). In comparison to *C. rangiformis*, the soil comprises a wide variety of different bacterial families, most prominent are representatives from the class of Actinobacteria (Fig. 4d). Within the phylum Pseudomonadota, the most prevalent are Alphaproteobacteria and in particular the family *Bradyrhizobiaceae*. In *C. rangiformis*, we found multiple families with comparable representation, among them *Acetobacteraceae* and *Caulobacteraceae* (Fig. 4c).

In addition, we found a strong enrichment of *Acidobacteriaceae* in the lichens in comparison to the corresponding soil. Out of the bacterial families that were found in lichens and soil, the families *Acidobacteriaceae*, *Acetobacteraceae*, and *Caulobacteraceae* had the highest abundance in absolute read numbers in the *C. rangiformis* lichen samples (Additional file 2: Fig. S12). However, the bacterial families *Capsulimonadaceae*, *Beijerinckiaceae*, and *Acidobacteriaceae* are most enriched in lichens when sequence read numbers of lichen and soil are compared (Additional file 2: Fig. S13). Most abundant with respect to absolute read counts in the soil are representatives of the families *Bradyrhizobiaceae* and *Pseudonocardiaceae* (Additional file 2: Fig. S12), the latter also showing one of the highest relative enrichments in the soil together with the families *Mycobacteriaceae*, *Nocardioidaceae*, and *Micromonosporaceae* (Additional file 2: Fig. S13).

Especially noteworthy is the presence of representatives of *Lichenibacteriaceae*, *Lichenihabitantaceae*, and *Halomonadaceae* exclusively in the *C. rangiformis* lichen, but with no incidence in the sampled soil. Furthermore, most of the taxonomic groups for which we could assemble bacterial genomes are strongly enriched in the lichen sample compared to the soil (Fig. 4c), indicating that their presence in the lichen might not be coincidental. However, more extensive sampling is needed to strengthen these observations. Further investigations must show whether some of these bacterial taxa are ecologically obligate symbionts of lichen thalli.

### Bacteria found in *C. rangiformis* represent a wide range of metabolic pathways

To study the metabolic potential of the microbial community found in *C. rangiformis*, we annotated metabolic subsystems in the identified metagenomic bins with RAST (Rapid Annotation using Subsystem Technology, rast.nmpdr.org). Of the 31 RAST categories, 27 were identified in our microbial sample, indicating that it contains a broad range of metabolic potential. Next, we compared the numbers of genes in the respective categories between our different metagenomic bins (Additional file 2: Fig. S14). BacD and BacN were the only ones that contain genes predicted to be involved in iron acquisition and metabolism. BacK stood out as the metagenomic bin with the most genes related to membrane transport and metabolism of aromatic compounds, while BacM contains genes involved in sulfur metabolism. Genes belonging to category photosynthesis were found only in BacF_chr2 (9 genes), BacK (8 genes), and BacO (8 genes). BacF was classified as belonging to the genus *Acidisphaera* of photosynthetic bacteria which was previously found in extreme environments such as acidic hot springs [32]. We speculate that the three photosynthetic bacteria play a supporting role in the photosynthetic activity of the lichen. We found none of the marker genes associated with nitrogen fixation, which is in agreement with previous work [83].

### Evidence for horizontal gene transfer between bacteria and algae and fungus

Because of the close physical proximity of the symbiotic partners in lichens, it is generally suggested that horizontal gene transfer (HGT) between them may be frequent, particularly between prokaryotic partners and fungi or algae. To identify putative HGT events, we searched for annotated genes in the *C. rangiformis* fungus and *A. mediterranea* algal chromosomes where Illumina reads mapped that had been classified by MEGAN as coming from bacteria (Additional file 2: Fig. S15). In this way, we identified a very strong HGT-candidate gene in *A. mediterranea* (Additional file 2: Fig. S15b). The gene *GR013A_c05g006500* encodes a protein that shows much higher similarity to bacterial proteins from NCBI (average 93%) than to algal proteins (average ~ 50%, Additional file 2: Fig. S15). In fact, one of the closest homologs comes from *Lichenicoccus roseus* (TLU73585.1), a species for which we could reconstruct a bacterial genome (see above). The genes neighboring the HGT candidate have highest similarity to proteins from *Trebouxiophyceae* algae, indicating a single gene transfer (Additional file 2: Fig. S15b). We emphasize that we found no indication of a sequence miss-assembly in that region, excluding the possibility of a technical artifact (Additional file 2: Fig. S15b). Additionally, the HGT candidate has a predicted novel exon at its 5’ end that has no homology in bacterial proteins.

The HGT candidate *GR013A_c05g006500* encodes a glutathione S-transferase (GST). GSTs are involved in a wide range of processes such as herbicide detoxification [24], cell signaling, plant development, and response to biotic and abiotic stresses [18, 20, 61, 73].

**Fig. 5:**
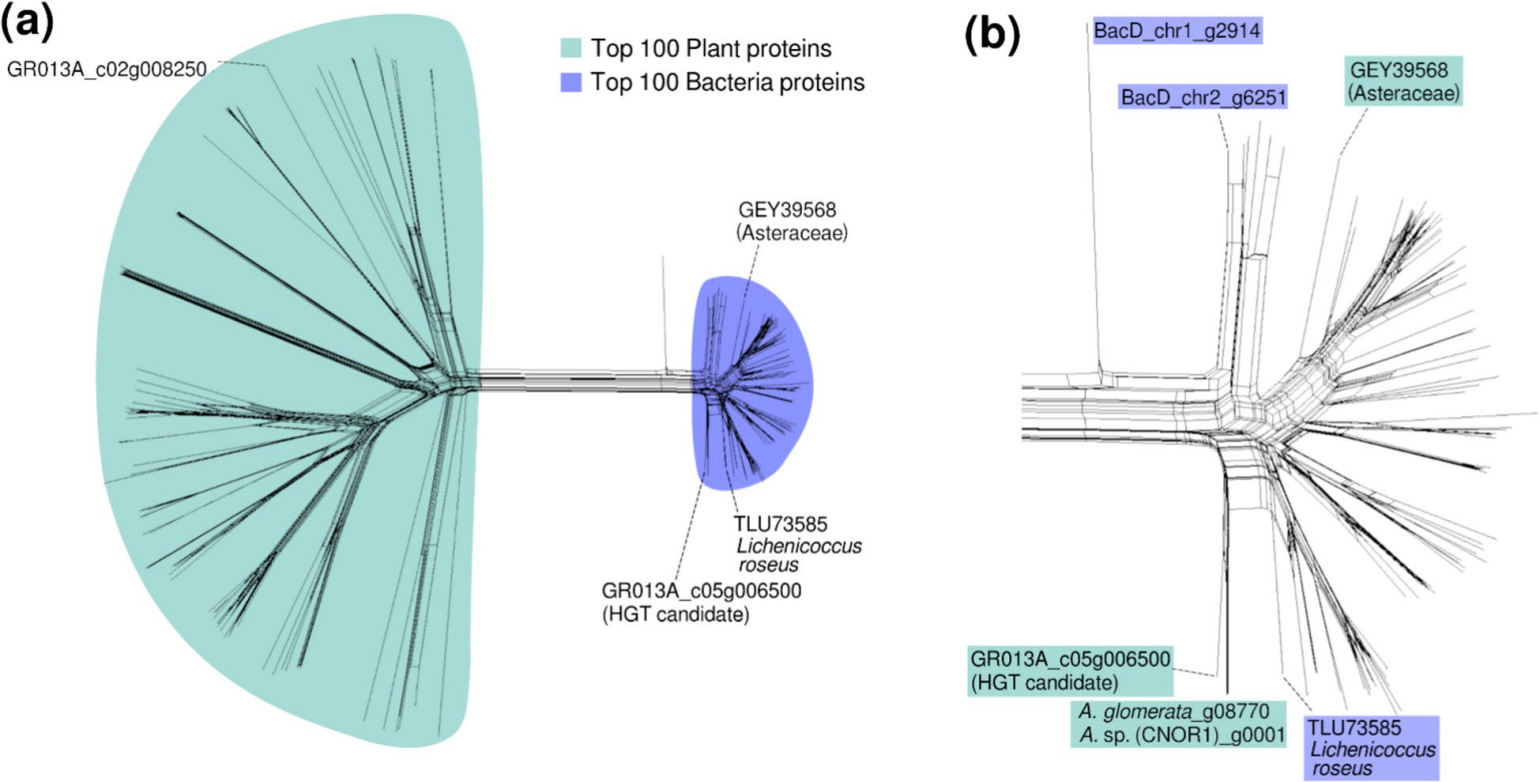
Split network of glutathione S-transferase (GST) proteins with the HGT candidate *GR013A_c05g006500* and identified plant and bacterial homologs calculated with the NeighborNet method. **a** Complete network with the top 100 plant and bacterial homologs. HGT homologs from Viridiplantae are marked in green, those from bacteria are shown in blue. **b** Zoom-in on the network of the 100 top bacterial homologs. The HGT candidate and its homologs from two publicly available *Asterochloris* genomes are shown in green. An additional homolog found in *Asteraceae* likely represents an independent HGT event. The closest bacterial homolog from NCBI (TLU7385) and those found in our bacterial genome BacD are shown in blue

To further validate our HGT candidate, we downloaded the 100 closest plant homologs from NCBI. The rationale was to determine whether plants have such homologs and whether they can be clearly distinguished from the bacterial proteins. We indeed found homologs from numerous plant taxa, including one in *A. mediterranea* (gene *GR013A_c02g008250*). We then produced a NeighborNet network that also included the top 100 bacterial homologs deposited at NCBI. The resulting network shows that the HGT candidate *GR013A_c05g006500* clusters with the bacterial proteins, while the ancestral plant homolog *GR013A_c02g008250* from *A. mediterranea* groups with other plant homologs (Fig. 5). Additionally, we did not find a *GR013A_c02g008250* homolog in published genomes of *Trebouxia* species. We conclude that plants generally have ancestral GST, but that *GR013A_c05g006500* was horizontally transferred possibly from a *Lichenicoccus* relative into *A. mediterranea* after the *Asterochloris* lineage separated from the *Trebouxia* lineage.

Surprisingly, the closest plant protein to GR013A_c05g006500 deposited at NCBI comes from the Dalmatian chrysanthemum (*Tanacetum cinerariifolium*), a flowering plant of the Asteraceae family. This protein also clusters with bacteria homologs, strongly indicating that this type of gene was transferred at least twice independently from bacteria to plants (Fig. 5).

Following the same method, we also identified two putative HGT events from bacteria into the fungus (Additional file 2: Fig. S16, S17, S18). However, the network tree indicates that both HGT events must have been ancient since both HGT candidates cluster close to bacteria, but together with other fungal proteins from several different taxonomic groups (Additional file 2: Fig. S17, S18). The two genes encode a methyltransferase domain-containing protein (*GR013F_c05g005660*), and a proline iminopeptidase-family hydrolase (*GR013F_c21g001150*).

### The *C. rangiformis* holobiont contains numerous gene clusters for secondary metabolites

Because secondary metabolites produced by lichens are of considerable biological and pharmacological relevance, we aimed to assess the potential for secondary metabolite biosynthesis within the lichen holobiont by identifying known biosynthetic gene clusters (BGCs).

**Fig. 6:**
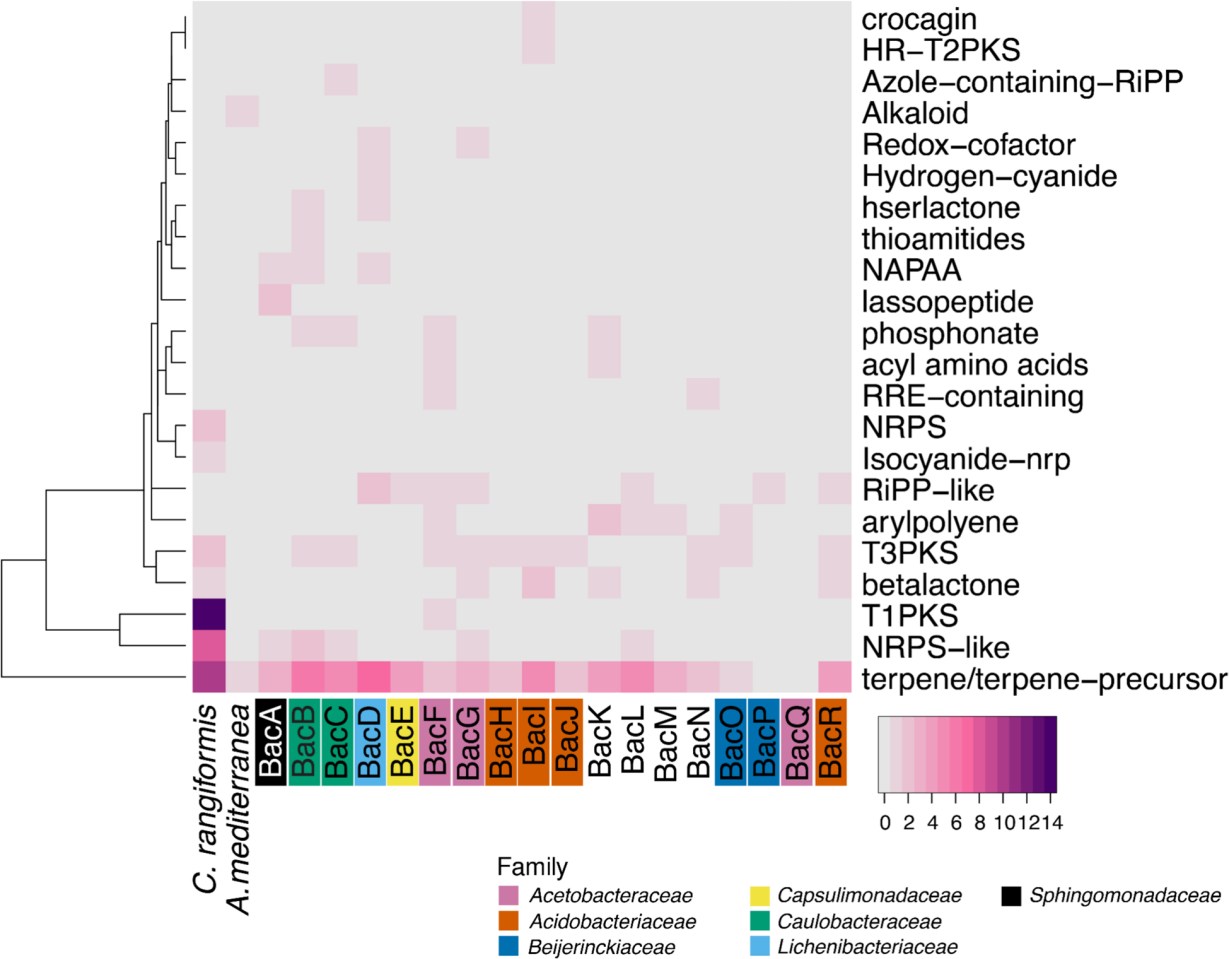
Biosynthetic gene clusters (BGCs) identified in the metagenome of *C. rangiformis*. BGCs were annotated using AntiSMASH. The heatmap is colored according to the number of identified clusters of a specific BGC category in each species (crocagin Crocagin-like; HR-T2PKS highly reducing type II PKS like ishigamide and skyllamycin; azole-containing-RiPP (Thio)azol(in)e-containing peptides, linear, and macrocyclic; hserlactone homoserine lactone; thioamitides thioamitide RiPPs as found in JOBF01000011; NAPAA non-alpha poly-amino acids like e-polylysin; RRE-containing RiPP recognition element containing cluster; NRPS non-ribosomal peptide synthetase; Isocyanide-nrp isocyanide with non-ribosomal peptide; RiPP-like other unspecified ribosomally synthesized and post-translationally modified peptide product (RiPP); T1/T2/T3PKS type I, II, and III polyketide synthase

Across the 20 species in our metagenomic dataset, we identified 22 distinct BGC categories (Fig. 6). The most abundant class was terpene-related BGCs (*n* = 70) which were found in all genomes, except in BacP and BacQ, with particular enrichment in the *C. rangiformis* mycobiont. This reflects recent findings of terpene biosynthetic genes being present in most, if not all, lichens [81]. However, the majority of BGCs were found only in one or a few genomes, such as those encoding crocagin and HR-T2PKS (Fig. 6).

In the mycobiont *C. rangiformis*, we detected BGCs with high similarity to known fungal BGCs. Notably, we identified clusters predicted to encode the biosynthesis of both grayanic acid (category T1PKS, Fig. 6), which is used as biochemical marker in lichen taxonomy [45] and 6-hydroxymellein (categories NRPS-like & T1PKS, Fig. 6), which was originally isolated from *Aspergillus terreus* and was shown to inhibit pollen development on Arabidopsis plants [77]. We also discovered BGCs with potential pharmacological importance. Among these was a cluster associated with the biosynthesis of FR901512 (categories T1PKS & NRPS, Fig. 6), an HMG-CoA reductase inhibitor with statin-like activity, known to reduce cholesterol synthesis [27]. Most notably, we identified a gene cluster encoding the production of clavaric acid (category terpene, Fig. 6), a compound that inhibits Ras protein activation and has demonstrated antitumor potential [39].

## Conclusions

To date, studies on lichen metagenomes are still rare, likely due to the complex task of isolating and analyzing genomes from multiple and diverse species. A recent study published the metagenome of the lichen *U. pustulata* [86]. However, that study focused on the assembly of fungal and algal genomes, while analysis of bacterial genomes was limited to rDNA. In contrast, 400 publicly available lichen metagenome sequences were re-examined, leading to the assembly of nearly 1000 MAGs [83], most of them from bacterial species.

Because of rapid technological development, the number of lichen metagenomes will grow in the future. Therefore, we considered it timely to assess and combine multiple different strategies to assemble high-quality MAGs for the fungus, alga, and bacteria. Here, we want to emphasize a few particularly successful approaches:

At a basic level, k-mer analysis proved highly effective in separating the main organism groups: fungus, alga and bacteria. This method also worked for much more fragmented short read assemblies of lichen metagenomes [69]. Additionally, because of their species-specificity and repetitive nature, TE sequences were highly effective to distinguish between plant and fungal sequences, and in some cases even between bacteria. To assemble chromosome-scale scaffolds for *C. rangiformis* and *A. mediterranea*, we also took advantage of recently published genome assemblies. Although from different species, chromosomes were sufficiently similar to anchor our sequence contigs. Furthermore, for assemblies of bacterial genomes, considering sequence read coverage was essential. Here, it proved particularly useful that we had metagenome data from additional various lichens. Our combined use of the correlation matrices as well as the software CONCOCT [4] and MetaBat [41] allowed reliable reconstruction of bacterial genomes, while use of only one of the approaches may not be sufficient. Ultimately, we succeeded in assigning ~ 80% of the ~ 220 Mb assembly to individual species.

Due to limited resources, the initial assembly was done on PacBio Sequel technology. It is conceivable that use of the latest long read technology combined with chromatin conformation capture (Hi-C) may produce less fragmented meta-genome assemblies. Nevertheless, our chromosome-scale assemblies are of comparable quality as recently published algal and fungal genomes that were produced from pure cultures (e.g., *C. grayi* and *A. glomerata*, [6]). However, while organelles assembled into single contigs, our bacterial MAGs are still fragmented to some degree.

The chromosome-scale assemblies allowed detailed insight into the organization of gene-containing and repetitive fractions of *C. rangiformis* and *A. mediterranea*, analyses that, to our knowledge, have not been done in lichens so far. We found both genomes to be highly compartmentalized regarding TE sequences. In *A. mediterranea*, we identified a pair of centromere-specific TEs that had strikingly resembling counterparts in grasses [31] where autonomous *RLG_Cereba* and non-autonomous *RLG_Quinta* retrotransposons are the main components of functional centromeres. Interestingly, the two systems must have evolved independently in algae and grasses, since *RLG_Beelzebufo* and *RLG_Cereba* represent different evolutionary lineages and contain different types of chromodomains, indicating that they acquired these domains independently.

The *C. rangiformis* fungus showed very strong RIP signatures, levels of which have been found in only a few fungi so far. RIP is proposed to occur during sexual reproduction [12]. The fact that we find only very few “unripped” TE sequences could mean that *C. rangiformis* frequently undergoes sexual recombination, despite clonal propagation being an important mechanism for the spread of symbiotic “propagules” [92]. Alternatively, *Cladonia* fungi may have TE silencing pathways additional to RIP.

Interestingly, we also identified putative HGT events from bacteria into the eukaryotic partners. However, it is notoriously difficult to prove HGT, especially if it occurred a long time ago and/or the prokaryotic gene donor is not known. Indeed, while some studies reported HGT [6], others found no evidence [86]. We were able to detect the origin of the GST which we regard as a likely horizontally transferred gene by, among others, relying on the annotation of proteins in the NCBI database. If proteins originating from assemblies of lichen (meta-)genomes are annotated as such and deposited in the NCBI database, a horizontally transferred protein will potentially have a higher similarity to proteins from other lichens than to the donor of the HT sequence, for example, bacteria, when using the blastp search against the NCBI database. We, therefore, expect that the detection of HGT events becomes increasingly difficult as more metagenomic resources for lichens become available. Additionally, we identified several notable BGCs, including those linked to the biosynthesis of grayanic acid, 6-hydroxymellein, and pharmacologically relevant compounds like FR901512 (a statin-like molecule) and clavaric acid (with antitumor potential). These findings highlight the lichen holobiont’s significant potential for producing bioactive secondary metabolites.

Our study was particularly informative with regard to the microbiome. The 18 bacteria species for which we assembled genomes were found at much lower abundance (or not at all) in surrounding soil, indicating they may be specifically proliferating in the lichen thallus.

However, to strengthen our conclusions, more extensive sampling is needed, as we do not have replicates of the lichen and soil samples in our current analysis. There are likely additional, less abundant species which we did not capture with our sequencing depth. Nevertheless, a recent study reported bacteria of the families *Acidobacteriaceae*, *Acetobacteraceae*, *Beijerinckiaceae*, and *Sphingomonadaceae* to occur most frequently in lichens [83]. These four families were also present in our 18 bacterial genomes. Additionally, all 18 were also found at least to some level in our 16 *Cladonia* samples coming from all over Europe. This indicates that the bacterial genomes isolated here are common and abundant cohabitants of lichens. Furthermore, we found two bacterial genera (*Lichenicoccus* and *Sphingomonas*) that were described in a previous study [83].

Particularly surprising were the presence of *Edaphobacter* and *Granulicella* species, previously described as soil bacteria adapted to arctic climate [42, 71]. We found them in consistent abundance in samples from north to south Europe. The two genera seem to be adapted to low carbon concentrations and metabolizing complex polysaccharides [42, 71]. These bacterial inhabitants of lichen thalli might mobilize carbohydrates from the glucan-rich fungal cell walls for their own nutrition.

At this point, it is still not clear which roles the bacteria play in the symbiosis and whether there are additional species which we did not detect due to low abundance. Contrary to our expectations, we found none of the marker genes associated with nitrogen fixation, as reported in a recent study [83], indicating that nitrogen fixation is not a typical feature of the microbiome of *C. rangiformis*. Moreover, we also found none of the previously described lichenicolous fungi [68]. However, three of the identified bacteria species have genes from photosynthesis pathways, suggesting that they may provide carbohydrate metabolites for the lichen symbiotic system.

Our *C. rangiformis* reference metagenome provides a glance at species diversity in a lichen symbiosis, but much broader research is needed for our understanding of the full complexity and interplay of the different taxa in the thalli of *Cladonia* species. Especially the role of individual bacteria species in the lichen symbiotic system is still obscure. In plants, mycorrhiza helper bacteria [26] and a wide range of other plant-beneficial bacteria have been characterized [85, 87]. Thus, with high probability, some representatives of the microbiome found in our lichen thallus are lichen-beneficial bacteria,their potential roles in the symbiosis remain to be explored.

## Methods

### Lichen sample collection

Lichen pieces were collected from various countries around Europe including Switzerland, Greece, Iceland, France, and Finland with all the sample collection details in Additional file 1: Table S2. We also collected a soil sample next to one lichen sample and have used this particular lichen as our reference sample (i.e., GR013).

### Metagenome sequencing and assembly

Individual lichen thalli were cleaned by gently swirling them in a paper towel lined funnel under running MilliQ water. Then, they were cleaned in a petri dish filled with MilliQ water using forceps to remove all remaining dirt, animal traces, and plant segments. The lichens were dried on paper towels and stored at − 20 °C.

High molecular weight DNA from lichen thalli was extracted using the previously published protocol for DNA extraction from wheat powdery mildew [11] with an additional purification step with magnetic beads [55], using SeraSil-Mag™ silica-coated superparamagnetic beads (Cytiva Europe GmbH,Freiburg im Breisgau, Germany added for impure samples (Additional file 1: Table S4). DNA from soil samples was extracted using the NucleoSpin(R) Soil kit from MACHEREY–NAGEL.

All samples were sequenced at the Functional Genomic Center at the University of Zurich (FGCZ) with Illumina technology. The lichen thallus samples were collected over the span of several years. Samples collected before 2020 were sequenced with 150 bp paired end reads, while the ones collected since 2020 were sequenced using a reads length of 250 bp (Additional file 1: Table S4). The Illumina DNA sequencing libraries were produced with the TruSeq DNA PCR-Free HT Kit to reduce the amplification bias. Additionally, the reference sample (GR013) of *C. rangiformis* was sequenced with PacBio technology, creating large insert libraries, and using the PacBio Sequel system and four SMRT Cells 1 M. The output reads were assembled by trying various assemblers (including SPAdes, flye, HGAP4, and megahit software) by the FGCZ. The assembly using Flye (v.2.6-g6f887ae) with “–meta” and “–pacbio-raw” parameters resulted in the least amount of contigs and overall best assembly. Next, the Arrow algorithm in SMRT Link was used on this assembly for further polishing. The Illumina reads were mapped back onto the assembly using bwa (v0.7.17) and pilon (v1.23) was run for final polishing (Additional file 1: Table S5). Illumina reads for all samples were also assembled with the metaSPAdes software (v3.13.1) at default parameters. These assemblies were used for a limited set of analyses, such as species definition using ribosomal DNA (rDNA, see below). RNA was also sequenced for sample GR004 using Illumina technology (Additional file 1: Table S4).

### Pre-processing the PacBio reference assembly

The Illumina reads of the reference genome were mapped onto the PacBio assembly using the Burrows-Wheeler Aligner (v0.7.17) [47]. We used the following functions: view, fixmate, sort, markdup, and index from the SAMtools package to further process the mapping [22, 48]. The mapping was used to subsequently calculate average read coverage of individual PacBio sequence contigs.

We screened the PacBio assembly for potential chimeric contigs (i.e., sequence contigs belonging to different species that were wrongly merged). Here, we calculated GC content over a sliding window of 200 bp to identify abrupt changes in GC content along contigs in order to distinguish, for example, between segments that come from bacteria (GC content > 50%) from segments that come from the fungus (GC content of < 50%). Additionally, fungal genomic sequences contain regions with very low GC content as a result of RIP. These were then examined by dot plot alignments of the sequence against itself which allowed narrowing down the boundaries between regions of high and low GC content (examples in Additional file 2: Fig. S1). With this method, we identified 9 chimeric contigs that joined fungal and bacterial segments. In two cases, the boundary could be determined precisely since it contained fungal telomeric repeats (multimers of CCCTAA), while in two cases, the boundary contains long poly C/G stretches. Chimeric contigs were then split by hand and the segments assigned either to fungal or bacterial contig groups.

### Assigning PacBio contigs to species groups

Frequencies of all 256 possible nucleotide tetramers were calculated for all PacBio sequence contigs larger than 10 kb. Shorter contigs were set aside because their tetramer frequency profiles are less representative. From this, a tetramer frequency matrix was generated which was then used for principal component analysis using R in order to separate the main species groups fungi, algae, and bacteria.

Contigs shorter than 10 kb were assigned to the main species groups based on transposable element (TE) sequences. TE sequences are highly species-specific and due to their repetitive nature can be used to assign genomic DNA segments to the respective species. Here, we first defined a reference set of large sequence contigs > 300 kb that were assigned to the alga and the fungus based on tetramer frequency, the presence of known plant and fungi-specific TEs, and genes with homology to previously published *Cladonia* or *Trebouxia* genomes (see Additional file 1: Table S1) but had no hits to contigs we defined as belonging to bacteria. This was done to exclude any contigs coming from bacteria that still may have ended up in the fungal or algal contigs. This resulted in a set of 43 high-confidence fungal and 32 algal contigs. We then used the < 10 kb contigs in blastn searches against the two reference datasets and identified all contigs with blastn hits > 300 bp. These hits were interpreted as coming from algal or fungal TEs, respectively, and the contigs assigned accordingly to alga or fungus contig pools.

Additionally, we searched the contigs shorter than 10 kb for RIP signatures. Here, we searched for sequences that had either an overall very low GC content of less than ~ 30%, and/or showed regions with abrupt changes from high to very low GC content. Of the 411 contigs identified in this approach, 403 were already assigned to the fungus in the TE search described above, which confirmed that low GC regions are mostly derived from fungal TEs. This step turned out to be largely confirmatory, since only 8 additional contigs were newly and uniquely identified through this approach to belong to the fungus.

Using tetramer frequencies for large contigs and the TE and RIP-based approaches for short contigs, we assigned 1420 contigs to the fungus and 198 to the alga pool (see Fig. 1 and S2).

### Assembly of chromosome-scale scaffolds for *C. rangiformis* and *A. mediterranea*

We used the recently published chromosome-scale assemblies of *C. squamosa* (Genbank accession GCA_947623385.2) and of an *Asterochloris* species (genbank accession GCA_963969365.1) as references to anchor PacBio contigs. Here, 1 kb segments at 10 kb intervals of the *C. squamosa* and *Asterochloris* reference chromosomes were used in blastn searches against the fungal and algal PacBio contig pool, respectively. This allowed identification of collinear segments that were then used to order the PacBio contigs into chromosomes. We were able to anchor 151 fungal and 55 algal contigs into chromosome-scale scaffolds. The 1269 fungal and 143 algal contigs that were not included in chromosome-scale scaffolds likely represent haplotypes and/or contaminants (Fig S2, see main text).

### Gene annotation

Fungal and algal genomes were annotated using the MAKER pipeline (v. 3.01.03; [14]). Repeats were masked prior to annotation by RepeatMasker (repeatmasker.org), employing the DFAM repeat database [82]. For evidence-based annotation of the *C. rangiformis* genome, we utilized protein and transcript data from *C. grayi* [6], fungal hydrophobin protein sequences obtained from NCBI, and EST data from *C. rangiferina* [40]. Evidence-based annotation of the algal *A. mediterranea* genome utilized protein data from *Trebouxia* sp. *A1-2* [86], and *A. thaliana* TAIR v10 proteins [9]. Additionally, a transcriptome assembly derived from *Cladonia mediterranea* RNA-seq data (comprising 219,316 contigs) was employed as EST evidence data. Initial sets of evidence-based gene models were utilized as input for the gene predictor SNAP (v2006-07–28; [44]) to construct hidden Markov model (HMM) profiles. Subsequent iterative refinement through multiple rounds of SNAP training produced the ultimate gene model sets.

Completeness of genome assemblies and predicted protein-coding gene sets was assessed using BUSCO (v. 5.7.1, [52, 80]) with the ascomycota_odb10 (2024–01–08) dataset for the fungal and the chlorophyta_odb10 (2024–01–08) dataset for the algal genome.

Reconstructed bacterial genomes (see below) were annotated with the RAST (rast.nmpdr.org) and Prokka [75], used for antiSMASH analysis, see below) pipelines. Completeness was assessed with BUSCO and CheckM (v1.1.6, [66], using the lineage workflow with the reduced tree option).

### Estimation of number of reads needed for assembly

PacBio long reads were mapped onto our reference genome using minimap2 [49]. The average read depth was then calculated using samtools depth for each bacterial MAG and compared to its respective BUSCO score (Additional file 2: Fig. S19).

### Species definition

Sequence assemblies of lichen samples were searched for contigs that contain ribosomal DNA (rDNA) genes. For fungi and algae, we extracted sequences which span short and conserved stretches from the 28S (at the 5’ end) both ITS and short stretches of 18S genes (at the 3’ end). The ITS sequences were then used as queries in blastn searches against NCBI. At least the top 20 BLAST hits were examined to account for potential misclassifications, as ITS-based species assignments do not always correspond with morphological identifications [58]. If blast hits were > 99% identical with the sequences from the predominant species in the top blast hits, it was assumed that our sample sequence was of that species.

Bacteria were classified based on 16S rDNA. Assemblies were searched with reference 16S sequences (e.g., from *E. coli* or *Burkholderia* species). Full-length 16S genes were extracted from the assemblies and used in blastn searches against NCBI. If the top hit was > 98% identical, the query was considered to be from the same species. Sequences with over ~ 95% identity were considered as belonging to the same genus.

### Phylogenetic analysis

rDNA sequences were aligned with ClustalW (v2.1) and phylogenetic analyses done with MrBayes (v3.2.7a) with settings nst = 6 and rates = invgamma [72]. The Markov chain Monte Carlo (MCMC) analysis was run until the average standard deviation of split frequencies decreased to under 0.01 with a sampling frequency of 10 (see Fig. 4), 100 and 200 and a burn-in of 25% of samples respectively. We chose to display the phylogenetic tree resulting from sampling frequency of 10 as we found virtually no difference in tree topology when using other sampling frequencies. Figtree (v1.4.4) (http://tree.bio.ed.ac.uk/software/figtree/) was used for visualization of phylogenetic trees.

### Reconstruction of bacterial genomes

For the further binning of the 937 bacterial contigs, we used MetaBAT2 [41], CONCOCT [4], and manual binning. For the manual binning, we mapped the Illumina reads of our 29 lichen samples onto our reference genome individually and calculated the sequence coverage of the contigs using the bamCoverage function from the package deepTools [70] from which we calculated average sequence coverage for each contig. From this, we combined values from all 29 samples into a single table using the R tidyverse library [91]. We prepared two tables using mapping data filtered by mapping quality 60 (mq60 and non-filtered reads (mapping quality 0 = mq0. From tables, we calculated correlation matrices which were clustered by hierarchical clustering utilizing the corrplot package (Version 0.92, [88]) and used them for visual binning.

To be considered a genome (i.e., a “metagenome bin”), a group of contigs was binned together by at least two of the four approaches. Since we used the rDNA for taxonomic classification, we applied here a more stringent threshold: contigs containing rDNA had to be binned together at least three binning methods, while simultaneously being binned into the same bin which represented the majority of contigs of the genome within the respective method. Bacterial metagenome bins were annotated with the RAST pipeline (rast.nmpdr.org).

### Comparison of gene content of fungal and bacterial genomes

Predicted proteins for the fungi *Letharia lupina*, *Ramalina farinacea*, *Xanthoria parietina*, *Blumeria graminis*, *Neurospora crassa*, and *Saccharomyces cerevisiae* were obtained from NCBI (ncbi.nlm.nih.gov/datasets/genome/). NCBI-conserved domains (CD) source files were downloaded from the NCI FTP site (ftp.ncbi.nlm.nih.gov/pub/mmdb/cdd). This included consensus sequences with functional descriptions as well as aligned protein sequences. The aligned conserved domains were converted into hidden Markov model (HMMs) with the hmmbuild program from the HMMER package (obtained from Ubuntu repositories, ubuntu.com). HMMs were converted into searchable databases with hmmpress (HMMER package). Protein datasets from fungi were searched against the HMM database with hmmscan (HMMER package). For each gene, all identifiers of CDs that had e-values > 10E-7 were extracted. To focus on gene families, only CDs that occurred at least 5 times in a given species were considered. Because multiple CDs may be hit by the same protein domains, they were grouped and gene descriptions were manually curated to identify common descriptions (e.g., “Ser/Thr kinases” or “Ankyrin repeat”). The curated descriptions were used to produce final counts for genes assigned to particular CD groups. Final CD counts from *C. rangiformis* were then compared pairwise to those of the other fungal species. Enrichment of CDs was tested with Chi-square tests. The -log 10 of the *p*-value was plotted against the log(2) of the ratio of CD numbers from *C. rangiformis* with those from the other fungal species. Volcano plots were produced with ggplot2 (R package).

For bacterial metagenome bins, gene categories provided by the RAST annotation (rast.nmpdr.org) were used to produce counts of genes belonging to respective categories. Counts for the individual categories across all bacterial metagenome bins. Category counts were visualized with the heatmap3 package from R.

### Biosynthetic gene cluster annotation

BGCs were predicted using antiSMASH using the bacterial, fungal, and plant-specific version for the respective organisms (v.8.0, [10]).

### Microbiome analysis

Illumina sequence reads were classified with the DIAMOND + MEGAN pipeline [8]. First, they were aligned to the ncbi nr databases using diamond (v 2.1.9) blastx. Weighted binning was done using the megan (v6.25.9) command daa2rma with the weighted lca algorithm. Read information was extracted with the command rma2info. For all microbiome analyses, we only used reads that were classified at least to bacteria family level, and focused on the 10 most abundant families. Utilizing the reads2class output, we extracted the read IDs associated with these 10 families (Fig. 4A and b). We then subsetted these reads from the mapping files utilizing the view function from samtools (v1.13) [22, 48], from which we counted reads per reconstructed bacterial genome for all 10 families.

For the heat trees in Fig. 4c and 4 d, comparing lichen and soil samples, we followed the “Analysis of Microbiome Community Data in R” instructions provided by the Grünwald Lab (github.com/grunwaldlab). For visualization, the node size range was set to between 1 and 5% of the graph’s width. The size range of the edges was set to 0.5% of the graph’s width. The packages metacoder (v0.3.6), taxa (v0.4.2), dplyr (v1.1.2), readr (v2.1.4), and ggplot2 (v3.4.2) were used. We obtained the necessary taxonomic classifications (kingdom, phylum, class, and order) from the NCBI Taxonomy database. All bacterial families with presence in either the lichen or the soil samples were included in the figures. For clarity, labels were removed for all families with less than 1% occurrence within the sample. For calculation of these percentages, only reads from families with complete taxonomy were used. Additionally, labels for other taxonomic levels were removed, if, e.g., represented families within an order all had an occurrence of under 1% in the sample each.

### Identification and analysis of HT candidates

To identify HGT candidates, we utilized the taxonomic classification of the Illumina short reads from the sample GR_013 we obtained from the DIAMOND + MEGAN. We extracted the names of the reads which were classified as bacteria from the reads2class output file and cross-matched them with the sam file output from mapping of the Illumina reads to our reference genome. We searched for reads that were mapped onto contigs that were integrated in fungal or algal chromosomes. Predicted genes that were covered by multiple reads were considered HGT candidates. Predicted proteins of the HGT candidates were used to search the NCBI conserved domains to determine their possible function. The HGT candidates were then used in blastp searches against the full NCBI database. If top hits were to bacterial proteins, we downloaded the top 100 sequences that were hit. To ascertain that eukaryotic homologs were more distant, we also downloaded the top 100 hits from blastp searches against the Fungi or Viridiplantae subdivision, respectively. Then, using the top 100 proteins from both searches, an alignment was produced using ClustalW (version 2.1, [46]). Alignments were cropped to the aligning part of the HGT candidate and re-aligned with ClustalW. Finally, a network was created using the NeighborNet algorithm with default settings in Splitstree4 (v4.19.2) [37].

To date the HGT event in *A. mediterranea*, we searched for homologs in the genomes of *Trebouxia gelatinosa* (PRJNA263654) and *T. lynnae* genomes (PRJNA1123266).

## Supplementary Information

Additional file 1: Table S1: Publicly available fungi and algae genome assemblies coming from lichens of the genus Cladonia. Table S2: Passport data of all the samples collected for this study. Table S3: Species definitions of the algal genomes described in this study based on sequence identity with publicly available ribosomal internal transcribed spacersequences. Note that some samples contain multiple high-scoring ITS sequences. Table S4: Details on the DNA/RNA extraction, library preparation and sequencing of all the samples that were sequenced in this study. Table S5: Statistics of initial assemblies of the reference metagenome sample Crans_GR013 using various assemblers. All statistics are based on contigs of size >= 500 bp, unless otherwise noted” and “Total length” include all contigs). Table S6: Classification of reconstructed bacterial MAGs based on 16S rDNA

Additional file 2: Figure S1: Identification and processing of chimeric sequence contigs. Figure S2: Workflow for the isolation of genomes from individual species in the reference metagenome assembly of *C. Rangiformis*. Figure S3: Analysis of the genomes of mitochondria from *C. rangiformis* and *A. mediterranea* and the chloroplast of *A. mediterranea*. Figure S4: Principal component analysisof k-mer frequencies in 1675 sequence contigs longer than 10 kb form the *C.rangiformis* reference metagenome assembly. Figure S5: Chromosome assembly and Analysis of gene content of *C. rangiformis*and *A. mediterranea*genomes. Figure S6: Identification of gene families that are specifically enriched in lichens Figure S7: GC content profiles from 112 ascomycete genomes Figure S8: Binning of PacBio sequence contigs into putative bacterial genomes Figure S9: BUSCO and CheckM scores of bacterial genomes. Figure S10: Test for presence of the bacteria identified in *C. rangiformis* in 29 lichen samples. Figure S11: MEGAN classified illumina reads which could be classified to the family level in the GR013 lichen sample. Figure S12: Proportional occurrence of bacterial families common to *C. rangiformis* lichen and corresponding soil samples. Figure S13: Enrichment factor of bacterial families found in both, the *C. rangiformis* lichen and its corresponding soil samples. Figure S14: Abundance of metabolic pathway genes in different metagenomic bins, as annotated by RAST. Figure S15: Identification of putative horizontal gene transfers. Figure S16: Distributions of sequence similarity [%] for algal and fungal proteins against bacterial proteins. Figure S17: Network of proteins from the NCBI database with the horizontal gene transfer candidate *GR013F_c21g001150*. Figure S18: Network of proteins from the NCBI database with the horizontal gene transfer candidate *GR013F_c05g005660*. Figure S19: Comparison of read coverage and BUSCO score of the bacteria species identified in the *C. rangiformis* metagenome.

## Data Availability

The scripts are deposited at (https:/github.com/Wicker-Lab/Cladoniaverse) [https://github.com/Wicker-Lab/Cladoniaverse] (https:/github.com/Wicker-Lab/Cladoniaverse). Genomic sequencing data was deposited in NCBI under the BioProject: PRJNA1168285.

## Abbreviations

AED: Annotation edit distance
BGC: Biosynthetic gene cluster
BUSCO: Benchmarking Universal Single-Copy Orthologs
CD: Conserved domains
CDS: Coding sequences
ChIP: Chromatine immuno precipitation
GC: Guanine-cytosine
GST: Glutathione S-transferase
HGT: Horizontal gene transfer
HMM: Hidden Markov model
ITS: Internal transcribed spacer
LTR: Long terminal repeat
MAG: Metagenome-assembled genome
OTU: Operational taxonomic unit
PCA: Principal component analysis
RAST: Rapid Annotation using Subsystem Technology
RIP: Repeat-induced point-mutation
TE: Transposable element
